# Mortality and Disability Due to Japanese Encephalitis in Elderly Adults: Evidence From an Adult Tertiary Care Center in West China

**DOI:** 10.3389/fneur.2019.00918

**Published:** 2019-08-22

**Authors:** Weixi Xiong, Lu Lu, Yingfeng Xiao, Jinmei Li, Dong Zhou

**Affiliations:** Department of Neurology, West China Hospital of Sichuan University, Chengdu, China

**Keywords:** Japanese encephalitis, elderly, mortality, follow-up, outcome

## Abstract

Japanese encephalitis (JE) is the most important cause of viral encephalitis in Asia, with most cases seen in children <15 years. Recently, cases of JE in people aged >50 years have been increasingly reported, but the clinical presentation in these cases is largely unknown. We report here the first case series of elderly JE patients from an adult tertiary hospital in West China. Medical records of laboratory-confirmed JE patients diagnosed from January 2011 to September 2018 were reviewed retrospectively. Patients were grouped into the elderly (patients > 50 years old) and control groups (patients aged 14–50 years). Data regarding demographics, clinical features, and outcome at discharge were collected. Telephonic follow-up was performed with the survivors in November 2018. Of the 50 patients with laboratory-confirmed JE, 11 were aged >50 years. In the elderly group, all patients had high fever and altered sensorium, and six had symptomatic seizures. Though the key symptoms as well as the cerebrospinal fluid and neuroimaging findings were similar in both groups, the worst Glasgow coma scale score was lower in the elderly group (6.14 ± 2.27 vs. 10.54 ± 3.37, *p* = 0.001). Compared to the control group, the incidence of acute secondary complications, including respiratory failure requiring mechanical ventilation or tracheotomy (81.82%), hypoalbuminemia (100%), thrombocytopenia (100%), deep venous thrombosis (63.64%), septicemia (36.36%), and upper gastrointestinal bleeding (27.27%) was higher in the elderly. The median modified Rankin scale (mRS) score at discharge was lower in the elderly group than in the control group (5 vs. 3, *p* = 0.017), with four and two cases of death, respectively. During the average 18-month follow-up, the median mRS score was 5 in the elderly and 2 in the control group (*p* = 0.001). Patients >50 years old accounted for 22% of JE cases diagnosed in a tertiary adult center, with high mortality rate and long-term disability compared to younger patients. Though no particular findings were found regarding clinical features and investigations in patients >50 years, most needed intensive care. In the future, it is imperative to recognize the importance of JE in adults and to reconsider the vaccination strategy in adult residents of endemic areas, especially for those over 50 years.

## Introduction

Japanese encephalitis (JE) is one of the most important forms of epidemic viral and vaccine-preventable encephalitis, with an estimated 68,000 cases and 15,000 deaths occurring yearly worldwide (1). Though JE was principally a disease of children, with 75% cases presenting in the age group under 15 years, several countries, such as South Korea and India, have seen a shift in the age distribution of JE (1–4). In South Korea, 89.9% (116 of 129) of JE cases between 2010 and 2015 were in patients aged more than 40 years; however, the clinical features and outcomes of the condition are not well-understood in the absence of a systematic study. It is important to understand the clinical features, mortality rates, and long-term outcomes of JE in elderly patients, such as patients over 50 years old, since some previous studies have suggested differences in certain features in adults and children with JE, such as higher mortality rates and better outcomes in adult patients (5, 6). In this retrospective case control study with follow-up, we aimed to gain further understanding of JE in patients over 50 years old.

## Methods

### Study Design

The electronic medical records of all patients diagnosed with JE in the West China Hospital between January 2011 and September 2018 were gathered for analysis. The West China Hospital is the largest tertiary adult hospital in Southwest China, admitting only individuals aged over 14 years. The diagnosis of JE was confirmed by the presence of JE virus-specific IgM antibody in the serum or cerebrospinal fluid (CSF), conducted and confirmed by the Chinese Center for Disease Control according to its standardized protocol (7). Patients were categorized into two groups, (1) the elderly group: patients who were 50 years or older when they were diagnosed with JE and (2) the control group: patients who were less than 50 years but over 14 years old when they were diagnosed with JE. In case of multiple admissions, patients in the subacute or chronic phase were excluded from the analysis.

### Clinical Information

Detailed disease-related information was collected from the medical records. Data regarding demographics, history of vaccination, history of chronic comorbidities, onset of the acute encephalitis, the precise day on which JE was confirmed, presence of high fever (>39°C), presence of altered consciousness, presence of witnessed seizures, presence of status epilepticus as per the criteria of the International League Against Epilepsy (8), length of hospital stay, cost, brain magnetic resonance imaging, or computed tomography findings, acute secondary complications, need for mechanical ventilation or tracheotomy and corresponding number of days were collected from the medical records. Elevation of CSF pressure was defined as pressure ≥180 mm H_2_O, elevated protein was defined as protein in the CSF ≥0.45 g/L, and pleocytosis of the CSF was defined as a CSF white blood cell count >8 × 10^−6^/L as per the normal hospital reference values. Similarly, hypoalbuminemia was defined as serum albumin below 55 gm/L and thrombocytopenia was defined as platelet counts below 100 × 10^9^/L. Glasgow Coma Scale scores were used to grade the severity, and the outcome at discharge was measured using the Modified Rankin scale (mRS) scores.

### Follow-Up

Follow-up of the survivors was conducted by one author (W.X.) by telephone in November 2018 by a standardized query of the present health status. The outcome mRS score was evaluated independently and blindly by two other authors (L.L. and Y.X.), and in cases of disagreement, a consensus was reached with opinion from a senior neurologist (J.L. or D.Z.).

### Statistical Analysis

Continuous variables are presented as means (± standard deviation) or median (interquartile range), and categorical variables are shown as numbers (%). SPSS version 19.0 for Windows (SPSS Inc, Chicago, IL, USA) was used for the analysis. Independent-sample *t*-test was used to assess the unadjusted differences in continuous variables between the two groups. Pearson chi-square test or Fisher's exact test was performed to compare the intergroup differences of categorical variables. Cost has been presented in RMB and $Intl (9). Follow-up data (mRS scores at follow-up, period of follow-up) of patients who died in the hospital were not included in the analysis. Significance was set at *p* < 0.05.

### Ethics Statement

This study was approved by the West China Hospital Medical Ethics Committee. Oral informed consent was obtained from the patients or their caregivers undergoing a telephonic follow-up.

## Results

### Demographics

After exclusion of cases with multiple admissions, 50 cases were identified from January 2011 to October 2018; in the years 2011, 2012, and 2014, there were no patients with JE in our hospital database. All patients had at least one positive laboratory test for JE. None of the patients had been tested for other viruses such as flaviviruses, including dengue virus, since Sichuan Province is inland and the number of cases reported is small; moreover, none of the patients in our study had a previous history of traveling abroad before onset of the disease. Eleven patients were over 50 years old (elderly group), and the remaining 39 patients were classified as the control group. In the elderly group, the median age of onset was 59 years (50 to 71 years), and seven were males. Seven of the elderly were Han people, two were Tibetan, one was Yi, and one was Man, all residing in Sichuan Province. All cases occurred between July and September. Seven patients denied being vaccinated for JE, and four were unable to provide precise information. Seven patients had history of chronic diseases, such as hypertension and diabetes; there was no evidence of any immune compromising condition. The number of comorbidities was significantly different between the two groups. No significant demographic differences were noted between the two groups (except for age; Table 1).

**Table 1:** Demographic and clinical features of the two groups with JE.

	**Parameters**	**Elderly group** **(*N* = 11)**	**Control group** **(*N* = 39)**	***p***
Demographics	Age (y)	59 (55,61.5)	27 (18,39)	3.78 × 10^−13^^**^
	Sex (F/M)	4/7	19/20	>0.05
	Han/Non-Han	7/4	31/8	>0.05
	No. of comorbidities	1.09 ± 1.04	0.26 ± 0.28	0.001^**^
Symptoms (No.)	Fever	11	39	>0.05
	Altered sensorium	11	36	>0.05
	Seizure	6	24	>0.05
	Status epilepticus	1	8	>0.05
CSF (No.)	Elevation of pressure	6	24	>0.05
	Elevated protein	11	35	>0.05
	Pleocytosis	10	32	>0.05
Neuroimaging (No.)	Insult of thalami, basal ganglia, midbrain, and other parts of the cortex	9	31	>0.05
	WMH	6	1	1.83 × 10^−4^^**^
Secondary complications (No.)	Required ventilator or tracheotomy	9	16	0.037^*^
	Hypoalbuminemia	11	29	>0.05
	Thrombocytopenia	11	7	1.09 × 10^−6^^**^
	DVT	7	3	3.05 × 10^−4^^**^
	Septicemia	4	2	0.017^*^
	UGB	3	7	>0.05

* p < 0.05;

** p < 0.01.

*JE, Japanese encephalitis; F, female; M, male; CSF, cerebrospinal fluid; WMH, white matter hyperintensities; DVT, deep venous thrombosis; UGB, upper gastrointestinal bleeding*.

### Clinical Features and Investigations

All patients were referred from other hospitals. All patients presented with the typical acute encephalitis syndrome (AES), featuring the acute onset of fever with altered sensorium and/or newly-developed seizures. While all patients presented with high fever (above 39°C) and altered sensorium, witnessed convulsions were present in six patients. Seizures were mostly controlled by a single anti-epileptic drug, except in one patient who developed focal status epilepticus with impaired consciousness, which was later controlled by an intravenous anti-epileptic drug. No statistically significant difference was noted between the elderly and control groups with regard to the clinical manifestations of AES (Table 1).

In the elderly group, six (54.55%) patients had elevated CSF pressures during the course of the disease. Protein elevation and pleocytosis of the CSF were observed in 11 and 10 patients, respectively. No difference was noted between the younger patients. Regarding the neuroimaging data, nine patients presented with hyperintensities in the thalami (7), basal ganglia (5), midbrain (1), or cortex (2) on brain magnetic resonance imaging. There were scattered small ischemic lesions seen as white matter hyperintensities in the white matter in six (54.55%) patients in the elderly group; these were not seen in the younger group (*p* = 1.83 × 10^−4^). Five patients in the elderly group and 28 patients in the control group had undergone lumbar puncture previously before being transferred to our hospital. However, JE testing was ordered in only three patients in the control group. The median duration to obtain a virology confirmation of JE was similar in the two groups (22.7 ± 11.29 days), ranging from 7 to 60 days.

As for the secondary complications, all but two elderly patients required tracheotomy due to respiratory failure, with a median length of ventilation of 26 days (9–33 days). Hypoalbuminemia and thrombocytopenia were observed in all patients, which required supportive treatment. Deep venous thrombosis in the lower limbs was seen in seven patients. Impaired liver function was present in four patients with slightly elevated alanine transaminase and/or aspartate transaminase (less than 200 IU/L) at some point in their disease. Septicemia was present in four patients, caused by bacteria including methicillin-resistant *Staphylococcus aureus, Acinetobacter baumannii*, and *Pseudomonas aeruginosa*; all patients needed intravenous antibiotics, and upper gastrointestinal bleeding was seen in three patients (Table 1). There was no evidence of renal failure as per the medical records.

### Outcomes

The individual worst Glasgow Coma Scale scores during the hospital stay and the mRS scores at discharge in patients over 50 years are listed in Table 2. Four patients died at discharge, and six were referred to secondary or rehabilitation hospitals for intravenous medication or because the patients were ventilator/catheter-dependent. Detailed information regarding cause of death at discharge is shown in the Supplementary Table; none of the patients underwent a post-mortem examination. The outcomes of the disease in the elderly and control groups are shown in Table 3; the worst Glasgow Coma Scale score was much lower in the elderly group compared to the controls. The mRS score at discharge also illustrated the trend. In the elderly group, the median mRS score was 5, and it was 3 in the control group.

**Table 2:** Severity of symptoms and outcomes of patients over 50 years old with Japanese encephalitis.

**Patient no**.	**Age**	**Sex**	**Ethnicity**	**GCS score at nadir**	**mRS score at discharge**	**mRS score at follow-up**	**Length of follow-up (m)**
1	50	M	Yi	5T (3+T+5)	4	6 (Died)	14
2	53	M	Han	5T (2+T+3)	5	5 (Mild cognitive impairment, altered personality, and bed-bound because of quadriplegia)	25
3	54	F	Han	5 (2+2+1)	5	2 (Mild cognitive impairment and hand tremor)	36
4	56	F	Han	6T (2+T+4)	5	5 (Severe cognitive impairment and bed-bound due to paralysis of the right upper limb and both lower limbs)	27
5	57	M	Man	6T (2+T+4)	5	4 (Mild cognitive impairment and paralysis of both lower limbs)	3
6	59	M	Han	3T (2+T+1)	4	2 (Monoplegia of upper limb)	15
7	59	M	Tibetan	died	6	–	–
8	61	M	Han	died	6	–	–
9	62	F	Han	died	6	–	–
10	66	M	Tibetan	died	6	–	–
11	71	F	Han	10 (3+3+4)	5	5 (Severe cognitive impairment and bed-bound because of severe diplegia)	3

*GCS, Glasgow coma scale; mRS, modified Rankin scale; M, male; F, female*.

**Table 3:** Outcomes of the two groups with JE.

**Parameters**	**Elderly group** **(*N* = 11)**	**Control group** **(*N* = 39)**	***p***
Interval from onset to JE diagnosis (d)	25.45 ± 12.45	21.92 ± 11.00	>0.05
Length of stay (d)	23.27 ± 13.67	25.97 ± 18.77	>0.05
Total cost (RMB)	77715.33 ± 49608.96	65316.65 ± 77156.28	>0.05
Daily cost (RMB)	3592.21 ± 1525.18	2147.67 ± 1158.55	0.001^**^
GCS score at nadir	6.14 ± 2.27	10.54 ± 3.37	0.001^**^
mRS score at discharge (No.)	11	39	0.017^*^
6	4	2	
5	5	14	
4	2	3	
3	0	8	
2	0	10	
1	0	2	
Period of follow-up (m)	18.57 ± 11.47	17.38 ± 13.07	>0.05
mRS score at follow-up (No.)	7	37	0.003^**^
6	1	0	
5	3	2	
4	2	2	
3	0	8	
2	2	10	
1	0	9	
0	0	6	

* p < 0.05;

** *p < 0.01*.

*JE, Japanese encephalitis; GCS, Glasgow coma scale; mRS, Modified Rankin scale*.

Daily cost, but not the length of stay or total cost, was significantly different between the two groups; the average daily cost in the elderly group was 3592.21 ± 1525.18 RMB ($Intl 1023.78 ± 434.68).

None of the patients were lost to the median 18-month follow-up (Tables 2, 3). One patient died because of respiratory failure within 1 month of discharge in the elderly group, and no one died in the control group. The median mRS score was 5 (range, 2 to 6) in the elderly group, which was significantly higher than that in the control group (median 2). None of the patients in the elderly group had a complete recovery. Four patients had severe sequelae including mild to severe cognitive impairment or paralysis requiring constant care. Two patients had moderate sequelae such as monoplegia that mildly affected function, but they were compatible with independent living. None of the patients in the elderly group reported seizures at follow-up, nor were they on medication (anti-epileptic drugs or drugs for symptomatic treatment).

## Discussion

Our study reports a series of 11 JE cases in people aged over 50 years, who accounted for 22% of the patients diagnosed with JE in an adult tertiary hospital. These patients had a relatively high mortality rate of 36.36% at discharge, and 45.45% during an 18-month follow-up, compared to the younger group. To the best of our knowledge, this is the first report to evaluate elderly patients with JE from an adult tertiary hospital in West China. Previous studies of mortality due to JE in adults reported varied rates, from 0 to 43%, with very few studies assessing the mortality in patients aged over 50 years (10–12). Studies that compared the demographics and clinical features between those with poor outcomes and better outcomes were unable to list age at onset as a risk factor, since these studies were performed in pediatric patients (2, 13). One retrospective study from South Korea identified 17 cases in three tertiary hospitals from 2010 to 2015; of these, 11 patients were bedridden at discharge, and 10 of the 17 patients were over 50 years old. Though the age of the patients did not have a significant association with whether the patients were bedridden or not, this result is explainable due to the small sample size of the study (10). Some studies have indicated that the mortality rate was higher as the age increased, with one study from China reporting a rate of 14.67% in patients over 70 years; this rate was relatively lower compared to that in our study, which might be due to the different study designs (14). Further studies of the mortality rate in patients aged over 50 years should be conducted based on the well-established national surveillance system with follow-up to avoid selection bias.

The clinical manifestations of AES, including fever, altered level of cognition, and seizures, were not different in elderly adults compared to those in younger adults according to our study. The CSF analysis did not reveal any significant difference in CSF protein or WBC levels, but in some studies that compared the CSF characteristics in adult and pediatric patients, differences were noted(2). No remarkable findings were found in the neuroimaging, except for the concurrent presence of white matter hyperintensities, which might be related to the pathomechanisms and vascular risk factors in elderly individuals, yet it was unknown whether it was irrelevant change before the acute onset of encephalitis. Regarding the neuroimaging findings of JE, thalamic lesions were noted in most patients in our study, and extrathalamic lesions such as lesions in the basal ganglia were also common; this was in accordance with previous studies (10). The most prominent problems during the course of the disease were not seizures or paralysis, but rather, secondary respiratory failure and other critical secondary complications which needed intensive care support. During the course of the disease, 9 out of 11 patients needed mechanical ventilation. Hypoalbuminemia, thrombocytopenia, deep venous thrombosis, septicemia, and upper gastrointestinal bleeding were also critical issues that required intervention; however, most of these conditions did not occur much in the control group. Thus, the likelihood of critical complications was higher in the elderly than in the younger age group.

The severe complications might also explain the higher daily cost in the elderly group.

Though there is no specific treatment for JE, timely diagnosis is still important for reducing unnecessary investigations and for evaluating the prognosis. The low rate of timely JE testing and the long duration from onset to diagnosis in the 50 patients indicate the lack of awareness and not envisaging the possibility of JE in adult patients, even in cases presenting with typical AES features in endemic areas and in particular seasons.

The outcomes were poor at discharge and on follow-up, with a mortality rate of 45.45% and disability rate of 100% in the elderly group. Previous reports have indicated that 50% of patients under 15 years develop neurological sequelae and behavioral disorders, but data of long-term outcomes of JE in patients aged over 50 years are scarce (12, 15). Some indicators of poor outcomes in children include elevated CSF pressure, repeated seizures, and SE; however, these were not remarkable in our cohort (12, 13). In contrast to an earlier study that indicated better prognosis in adults than in children, our preliminary results showed the possibility that patients aged over 50 years might be an independent risk factor of poor prognosis; further multicenter case-control studies with larger populations are required to confirm this (5).

JE is the most common cause of viral encephalitis in China, and thousands of cases were reported annually earlier. Though the vaccine has been available in China since the 1960s, only few individuals opted to get vaccinated, as the cost of JE vaccination was borne by the individual from the 1960s to 2000 (16). From 2008, China initiated the National Expanded Program on Immunization schedule to vaccinate infants. The recommendation for children is vaccination at 8 months and 2 years of age with live, attenuated SA 14–14–2 JE virus vaccine, and JE cases in China have dropped significantly in the last decade (17). However, most adults were not inoculated with the vaccine and might be vulnerable to JE. The number of patients with JE aged over 15 years has been on the rise in the last few years, accounting for 82.48% of the total patients in 2016, and for the first time, the number of patients aged between 15 and 50 years outnumbered those under 15 years of age. The proportion of patients over 50 years with JE is also on the rise, increasing from 2.93, 3.42, and 2.36 during 2004–2006 to 14.77, 5.51, and 6.97% during 2013–2015, and the figure was shockingly high in 2016 (21.82%) (18). Adults have been reported to be more vulnerable with a higher mortality rate in an outbreak (19, 20); however, elderly patients were not given enough attention, as senior groups were not listed in the special risk groups in the World Health Organization's position paper on JE (21).

Our study has some limitations. The first limitation is the small sample size of the studied population; it is possible that selection bias may have existed since this study was based on data from a tertiary adult hospital, with all patients referred from other hospitals; hence, our results related to the proportion, severity, and mortality cannot be generalized to all JE patients over 50 years old. A second limitation was the retrospective design, wherein data such as residential information and usage of bed nets was not available and patients were unable to be examined due to the same period after their discharge. Thirdly, we only performed telephonic follow-up; physical examination was not performed, as it was inconvenient for patients with sequelae. However, some studies have shown that telephonic assessment of mRS scores is also reliable and comparable to direct face-to-face assessment (22–24).

Despite these limitations, our report showed that patients over 50 years old comprised a considerable proportion of patients with JE and had a high mortality rate and long-term disability. All patients presented with typical AES and most needed intensive care due to respiratory failure and other critical secondary complications. In conclusion, physicians should be aware of the high incidence of JE in patients over 50 years, and quick diagnosis is essential, which can lead to appropriate management in the early stage. Furthermore, given that JE is a vaccine-preventable disease, consideration should be given to reframing the vaccination strategy for people residing in endemic areas, especially in the elderly population.

## Data Availability

The raw data supporting the conclusions of this manuscript will be made available by the authors, without undue reservation, to any qualified researcher.

## Ethics Statement

This study was carried out in accordance with the recommendations of STROBE with oral informed consent from all subjects with follow-up. All subjects undergoing a telephonic follow-up gave oral informed consent in accordance with the Declaration of Helsinki. The protocol was approved by the West China Hospital Medical Ethics Committee. For subjects without follow-up, exception to the requirement of informed consent was proved by the West China Hospital Medical Ethics since data was obtained from the Electronic Medical Record.

## Author Contributions

WX and DZ contributed to the conception and design of the study. WX, LL, and YX conducted the follow-up. WX performed the statistical analysis and wrote the first draft of the manuscript. LL, YX, and JL wrote sections of the manuscript. All authors contributed to manuscript revision, read, and approved the submitted version.

### Conflict of Interest Statement

The authors declare that the research was conducted in the absence of any commercial or financial relationships that could be construed as a potential conflict of interest.

## Abbreviations

AES: Acute encephalitis syndrome
CSF: Cerebrospinal fluid
JE: Japanese encephalitis
mRS: Modified Rankin score.
