# Supplementary material for: Mortality and Disability Due to Japanese Encephalitis in Elderly Adults: Evidence From an Adult Tertiary Care Center in West China
Source: Front Neurol. 2019 Aug 22;10:918. doi: 10.3389/fneur.2019.00918 (PMC6714058; doi:10.3389/fneur.2019.00918)
Supplement: Supplementary file 1 [file Table_4.DOCX]

Supplementary Material

**Supplemental table** Causes of death at discharge

| Patient No. | Age | Cause of death | Disease duration(day) |
| --- | --- | --- | --- |
| 7 | 59 | Respiratory failure and uncontrolled septicemia | 5 |
| 8 | 61 | Uncontrolled septicemia | 28 |
| 9 | 62 | Respiratory failure and uncontrolled septicemia | 10 |
| 10 | 66 | Respiratory failure | 22 |
| 34 | 25 | Respiratory failure | 19 |
| 37 | 20 | Respiratory failure and uncontrolled septicemia | 12 |

None of the patients had a post-mortem examination.
